# Knowledge and Expectations of Hearing Aid Apps Among Smartphone Users and Hearing Professionals: Cross-sectional Survey

**DOI:** 10.2196/27809

**Published:** 2022-01-07

**Authors:** Jae Sang Han, Yong-Ho Park, Jae-Jun Song, Il Joon Moon, Woojoo Lee, Yoonjoong Kim, Young Sang Cho, Jae-Hyun Seo, Moo Kyun Park

**Affiliations:** 1 Department of Otolaryngology-Head and Neck Surgery College of Medicine The Catholic University of Korea Seoul Republic of Korea; 2 Department of Otolaryngology-Head and Neck Surgery College of Medicine Chungnam National University Daejeon Republic of Korea; 3 Brain Research Institute College of Medicine Chungnam National University Daejeon Republic of Korea; 4 Department of Otorhinolaryngology-Head and Neck Surgery Korea University College of Medicine Seoul Republic of Korea; 5 Department of Otorhinolaryngology-Head and Neck Surgery Samsung Medical Center Sungkyunkwan University School of Medicine Seoul Republic of Korea; 6 Department of Public Health Sciences Graduate School of Public Health Seoul National University Seoul Republic of Korea; 7 Department of Otorhinolaryngology, Head & Neck Surgery Seoul National University Hospital Seoul National University College of Medicine Seoul Republic of Korea; 8 Sensory Organ Research Institute Seoul National University Medical Research Center Seoul Republic of Korea

**Keywords:** smartphone, hearing aids, app, perception, survey, hearing loss, mobile phone

## Abstract

**Background:**

Despite the increasing prevalence of hearing loss, the cost and psychological barriers to the use of hearing aids may prevent their use in individuals with hearing loss. Patients with hearing loss can benefit from smartphone-based hearing aid apps (SHAAs), which are smartphone apps that use a mobile device as a sound amplifier.

**Objective:**

The aim of this study is to determine how ear, nose, and throat outpatients perceive SHAAs, analyze the factors that affect their perceptions, and estimate the costs of an annual subscription to an app through a self-administered questionnaire survey of smartphone users and hearing specialists.

**Methods:**

This study used a cross-sectional, multicenter survey of both ear, nose, and throat outpatients and hearing specialists. The questionnaire was designed to collect personal information about the respondents and their responses to 18 questions concerning SHAAs in five domains: knowledge, needs, cost, expectations, and information. Perception questions were rated on a scale of 1 (strongly disagree) to 5 (strongly agree). Questions about the expected cost of SHAAs were included in the questionnaire distributed to hearing experts.

**Results:**

Among the 219 smartphone users and 42 hearing specialists, only 8 (3.7%) respondents recognized SHAAs, whereas 18% (47/261) of respondents reported considering the use of an assistive device to improve their hearing capacity. The average perception score was 2.81 (SD 1.22). Among the factors that shaped perceptions of SHAAs, the needs category received the lowest scores (2.02, SD 1.42), whereas the cost category received the highest scores (3.29, SD 1.14). Age was correlated with the information domain (*P*<.001), and an increased level of hearing impairment resulted in significantly higher points in the needs category (*P*<.001). Patients expected the cost of an annual app subscription to an SHAA to be approximately US $86, and the predicted cost was associated with economic status (*P*=.02) and was higher than the prices expected by hearing specialists (*P*<.001).

**Conclusions:**

Outpatients expected SHAAs to cost more than hearing specialists. However, the perception of the SHAA was relatively low. In this regard, enhanced awareness is required to popularize SHAAs.

## Introduction

Hearing loss is one of the most common health care problems worldwide. When the World Health Organization started reporting hearing loss in 1985, the number of people with moderate-to-profound hearing impairment was estimated to be 42 million. Furthermore, the number of people with disabling hearing loss reached 466 million in 2018 and is projected to reach approximately 630 million by 2030 [1]. Hearing aids (HAs) are standard hearing intervention methods [2], and the adequate use of HAs improves hearing-specific and general health–related quality of life in adults with mild to moderate hearing loss [3].

Nevertheless, HA adoption rates are extremely low. Globally, only 17% of those who need appropriate hearing rehabilitation use HAs [4]. In addition, a large South Korean cohort study reported that among participants who had minimal hearing loss (mild bilateral hearing loss, unilateral hearing loss, and high frequency hearing loss), only 0.47% of those with subjective symptoms used HAs [5]. Failure to achieve early rehabilitation can accelerate the development of hearing loss and, ultimately, incur enormous social costs [6]. The price of HAs is an important barrier to use [7,8]. When hearing health care is subsidized by the government, HA penetration rates slightly increase [9]. Therefore, other factors, such as social stigma, denial of hearing loss, reduced self-efficacy, and limited access to hearing services should be considered [8]. A prolonged time from the onset of hearing loss to HA intervention has negative effects on quality of life [10]. To address these barriers, alternatives such as over-the-counter (OTC) HAs, personal sound amplification products (PSAPs), and smartphone-based HA apps (SHAAs) have been previously evaluated [11-14]. Moreover, the US Food and Drug Administration announced in its 2016 nonbinding guidance document that medical assessment is no longer required for OTC HAs for individuals aged ≥18 years [15].

SHAAs were originally developed to mimic conventional HA devices. SHAAs refer only to the software installed on a mobile device for hearing support, which is different from the traditional HA hardware–software complex. SHAAs require wired or Bluetooth headsets or headphones instead of hardware resources. Many free or low-price HA apps are available on the web. Although they enhance hearing capabilities through sound amplification, SHAAs were previously far less sophisticated because they could not exactly fit an individual’s prescribed target gain as could HAs fitted using real-ear measurement [16]. Some SHAAs have separate channels and advanced functions, such as noise reduction and acoustic feedback suppression [17]. Until now, it was not clear whether SHAAs were clinically effective and could be an alternative device to traditional HAs [18]. In addition, their level of patient satisfaction is generally lower than that with conventional HAs [19]. However, the performance of SHAAs is likely to improve with the development of smartphone hardware and apps, and SHAAs have great potential to contribute to hearing rehabilitation [20].

Easy accessibility is a notable advantage of SHAAs. Users can simply download the app on their smartphones and prepare headsets or headphones for use. SHAAs may particularly help overcome psychological resistance to the use of HAs. Trials with SHAAs showed a reduction in the degree of anxiety and personal distress and increased self-esteem. In addition, reduced stigma or body image of HA users can be expected because of the growing number of individuals who wear headphones with their smartphones [21]. Maidment et al [22] demonstrated that the use of smartphone-connected listening devices in adults with hearing loss could address issues surrounding stigma because smartphones are ubiquitous in everyday life. In addition, the price of SHAAs is lower than that of conventional HAs or PSAPs [23]. Dozens of SHAAs have been released in the App Store (iPhone operating system) and Google Play (Android). Moreover, a new SHAA called Sound Amplifier was introduced by Google [24,25].

The mobile app market is rapidly growing. As of 2019, about 61% of the global population was able to access the internet from mobile devices, and this number is projected to increase to approximately 79% by 2025 [26]. Furthermore, as an increasing number of older adults (>65 years) are using mobile internet via their smartphones, smartphones are expected to exert a greater influence on hearing health care, and SHAAs will expand accordingly [20,27]. Nevertheless, no previous studies have focused on how SHAAs are perceived and the factors affecting the perception of SHAAs. Thus, in this study, we assessed the current awareness of SHAAs and analyzed the associated factors through questionnaires. This information will serve as a baseline for further research on hearing rehabilitation using SHAAs.

## Methods

### Participants

We performed a multicenter survey of 5 general hospital outpatients who use smartphones and hearing specialists, including otology specialists, audiologists, and HA researchers. Before gaining access to a questionnaire, the potential participants were informed about the survey, and those who agreed to participate were asked to fill out the questionnaire under the direction of a health care provider.

This study was carried out in accordance with the Declaration of Helsinki on biomedical research for human participants, and the study protocol was approved by the institutional review board of each participating hospital (Seoul St Mary’s Hospital, KC20QIDI0526; Chungnam National University Hospital, 2020-06-092; Korea University Hospital, 2020GR0020; Samsung Medical Center, 2020-05-056; and Seoul National University Hospital, D-2003-028-1109).

### Questionnaire

A survey on the perception of HAs published by Park et al [28] was modified for use in this study because there is no standardized questionnaire available to assess perceptions of HAs, including SHAAs. Park et al developed a questionnaire that contained 19 questions with an appropriate level of reliability and validity (Cronbach α=.76). To evaluate the consistency of questionnaire items, Hotelling T-square test was used. The items had significant reliability, with *F*=28.5, *P*<.001 [28]. One question (“I know that different types of HAs can be worn depending on the degree of hearing loss”) was excluded from the questionnaire by Park et al because it was not suitable for the SHAA questionnaires. In addition, *hearing aids* was replaced with *smartphone-based hearing aid apps*. A total of 18 questions in the questionnaire were reviewed by 42 hearing rehabilitation specialists who participated in the opinion survey, and Cronbach α for each question was recalculated (Multimedia Appendix 1). The language used in the questionnaire was Korean. To prevent any possible confusion, respondents were fully informed that SHAAs are independent substitutes for HAs and do not require an additional device other than a smartphone and headphone or headset.

The questionnaire was divided into three sections: (1) sociodemographic characteristics, including age, gender, residence, educational background, economic status, and occupation; (2) clinical characteristics, including the recognition of hearing loss and inconvenience level, the presence of tinnitus and inconvenience level, previous experience with PSAPs or SHAAs by the respondent or their family member, respondent’s willingness to use PSAPs or SHAAs, and expected cost of the app; and (3) perception status. In the clinical characteristics section, respondents with hearing loss or tinnitus were asked to assess the degree of their symptoms using a visual analogue scale (VAS). In the perception status section, they were asked to rate 18 questions in 5 categories on a scale from 1 (strongly disagree) to 5 (strongly agree); lower scores indicate poorer awareness. The questions were grouped into 5 categories by similar objectives, which were reviewed by the hearing specialists, allowing the analysis to be simpler and clearer. Questions 1-4 were grouped in the knowledge category, which aimed to evaluate whether respondents were aware of SHAAs as hearing rehabilitation options and how they differed from conventional HAs. Questions 5-6 in the needs category were designed to evaluate whether respondents thought that SHAAs were necessary for hearing discomfort. Questions 7-9 in the cost category were used to identify the influence of price on the decision to purchase, and questions 10-13 were used to evaluate respondents’ expectations regarding the ability of SHAAs to improve hearing capabilities. Finally, questions 14-18 in the information category attempted to determine whether participants had accurate information about how to use SHAAs (Multimedia Appendix 1).

The opinion survey for hearing specialists contained questions to determine demographic information such as employment history, educational background, and professional experience (length of career) as well as the expected annual subscription rate for an SHAA and the main selection criteria for HA devices (Multimedia Appendix 2).

Korean Won was used as the standard currency in the questionnaire and was converted into US $ in this report (US $1=₩1082.50).

### Statistical Analysis

Age, gender, education background, and economic status data were treated as categorical variables. Reference variables were 20-39 years for age, male for gender, middle school graduate for educational background, and 1 for economic status. VAS score of hearing loss and VAS score of tinnitus were regarded as continuous variables. Linear regression models with the perception level and the expected annual subscription rate as response variables were applied. Robust variance estimation was used for SEs and CIs. Age, gender, educational background, economic status, VAS score of hearing loss, and VAS score of tinnitus were used as explanatory variables in the regression models. Bonferroni-corrected *P* values <.05 were considered statistically significant. The 2-sample 2-tailed *t* test was used to compare the expected costs between the hearing specialists and potential users. All statistical analyses were performed using R version 3.6.0 (R Foundation for Statistical Computing).

## Results

### Clinical Characteristics of Enrolled Participants

A total of 98.6% (219/222) of respondents’ answers were analyzed after the survey responses of 3 participants with a survey completion rate <50% were excluded.

The clinical characteristics of the participants are presented in Table 1. The mean age of participants was 52.02 (SD 15.44) years, and male respondents slightly outnumbered female respondents at 59.8% (131/219 male respondents) to 40.2% (88/219 female respondents). Most respondents (138/219, 63%) were college graduates or higher education, and about half (114/219, 52.1%) of the participants estimated themselves as having an intermediate economic status. A total of 44.3% (97/219) of respondents answered that they had subjective hearing loss. The average VAS score of respondents with hearing loss was 2.51 (SD 3.29). In addition, 40.2% (88/219) of respondents had tinnitus, and their average VAS score was 2.39 (SD 3.32). Owing to the multicenter nature of the study, the locations of the participants’ residences varied widely. Most participants lived in urban areas (130/219, 59.3%), followed by suburban areas (59/219, 26.9%) and rural areas (30/219, 13.7%). Only 0.9% (2/219) of the enrolled participants had been using HAs at the time of the survey, so wearing HAs was not used in the analysis.

When asked about SHAAs, 21.5% (47/219) of respondents stated that they had considered using an assistive device for hearing loss, but only 3.7% (8/219) respondents knew the difference between traditional HAs and SHAAs. Only 0.9% (2/219) of respondents had experience with an SHAA. However, 26.5% (58/219) of respondents expressed a willingness to use an SHAA in the future.

A total of 42 responses were received from the hearing specialist group, which comprised 29 (69%) otologists and 13 (31%) audiologists. The average number of years working in this profession was 11.83 (SD 7.81) years. A total of 40% (17/42) of respondents had a bachelor’s degree, followed by 33% (14/42) of respondents with a master’s degree and 26% (11/42) of respondents with a doctorate degree.

**Table 1:** Participant characteristics (N=219).

Characteristics		Value
Age (years), mean (SD)		52.0 (15.4)
**Sex, n (%)**		
	Male	131 (59.8)
	Female	88 (40.2)
**Education level, n (%)**		
	Junior high graduate or less	29 (13.2)
	High school graduate	53 (24.2)
	College graduate or higher	137 (62.6)
**Economic status, n (%)**		
	A (very low)	12 (5.5)
	B (low)	29 (13.2)
	C (middle)	113 (51.6)
	D (high)	52 (23.7)
	E (very high)	13 (5.9)
**Subjective hearing loss, n (%)**		
	Yes	97 (44.3)
	No	122 (55.7)
If hearing loss “yes,” VAS^a^ score (1-10)^b^, mean (SD)		2.5 (3.3)
**Tinnitus, n (%)**		
	Yes	88 (40.2)
	No	131 (59.8)
If tinnitus “yes,” VAS score (1-10)^c^, mean (SD)		2.4 (3.3)

^a^VAS: visual analogue scale.

^b^Visual analogue scale (VAS) 1=very minimal problem; VAS 10=very serious problem. VAS 0 was considered to indicate no subjective hearing loss.

^c^Visual analogue scale (VAS) 1=very minimal problem; VAS 10=very serious problem. VAS 0 was considered no subjective tinnitus.

### Overall Awareness of SHAAs

**Figure 1:**
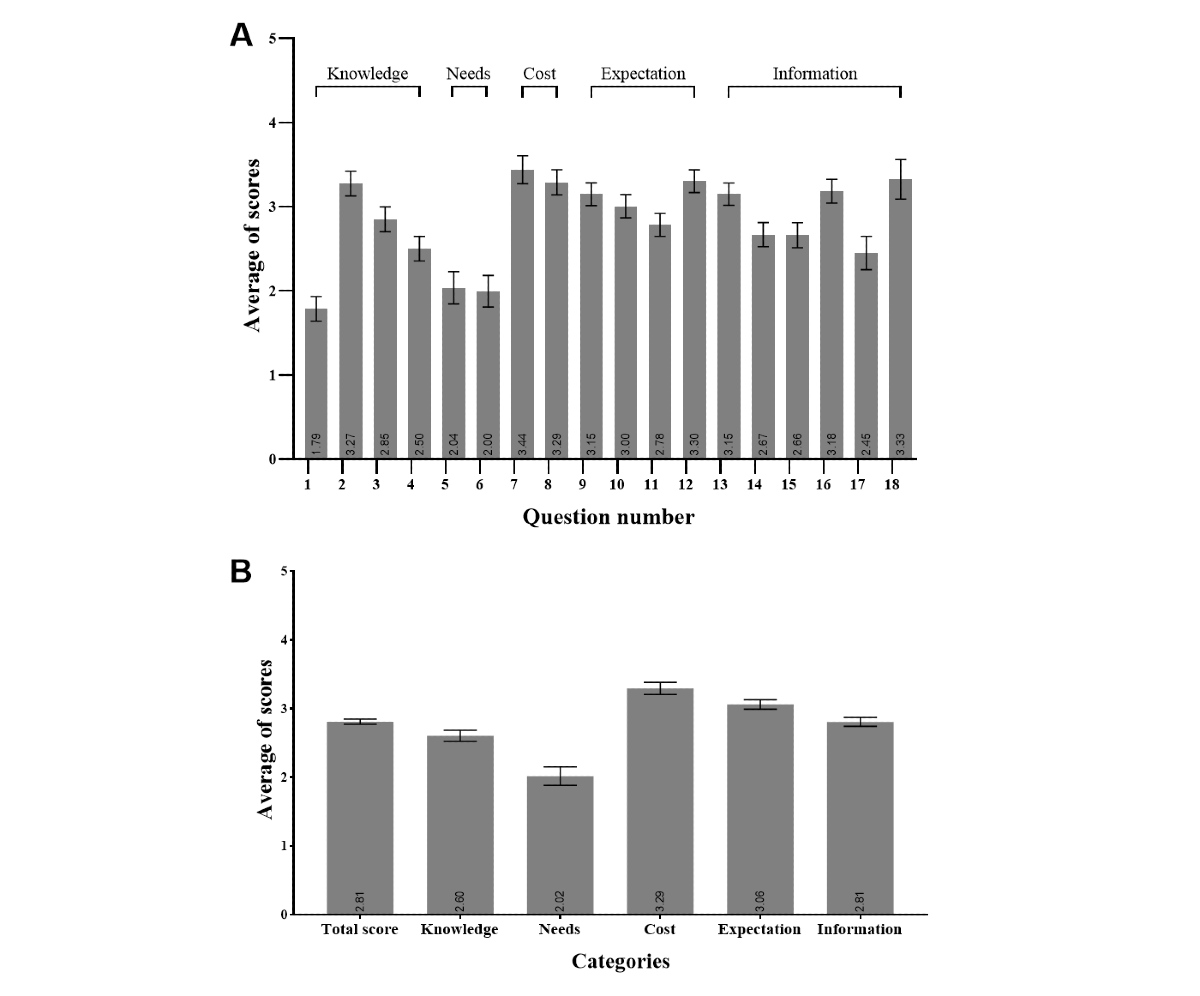
Average scores obtained in the perception survey. (A) Average scores of 18 questions (rated by question). (B) Average scores of 5 categories (rated by category). Error bars indicate 95% CIs.

The overall score of awareness of SHAAs of the 219 respondents was 2.81 (SD 1.21). Among the 5 categories, the needs category received the lowest score of 2.02 (SD 1.42), whereas the cost category ranked first with a score of 3.29 (SD 1.14; Figure 1).

In the opinion survey of hearing specialists, the main consideration factor for recommending an SHAA was basic performance (30/42, 71%), followed by price (8/42, 19%) and additional functions (2/42, 5%). In addition, noise reduction and the number of channels were mentioned by 1 respondent each.

### Factors Affecting Awareness Scores

#### Age, Gender, and Area of Residence

Respondents were divided into three age groups: 20-39 years, 40-59 years, and ≥60 years. Compared with the reference age group of 20-39 years, there was no association between age and perception scores in the 40-59–year group, although there was a marginally significant positive correlation between age and information score (*P*=.05) in this age group. Meanwhile, in the ≥60-year group, there was a remarkable positive correlation between the total SHAA perception score and age in comparison with the 20-39–year group (*P*=.002), and there were also strong associations between the information and perception scores among the 5 categories (*P*<.001; Table 2).

To analyze whether gender affected SHAA perception, male respondents were used as the reference group. There were no significant correlations between gender and SHAA perception (Table 3). The area of residence was also not significantly correlated with SHAA perception.

**Table 2:** Relationship between age and perception scores on smartphone-based hearing aid apps. The reference age group was the 20-39–year group.

Response			Coefficient (SE; 95% CI)		*P* value		Adjusted *P* value
**40-59–year group**			0.089 (0.095; −0.097 to 0.276)		.35		N/A^a^
	Knowledge	−0.155 (0.131; −0.411 to 0.102)		.24		.99	
	Needs	0.209 (0.155; −0.094 to 0.511)		.18		.89	
	Cost	0.007 (0.168; −0.323 to 0.336)		.97		.99	
	Expectation	−0.003 (0.148; −0.293 to 0.288)		.99		.99	
	Information	0.301 (0.118; 0.069 to 0.533)		.01^b^		.05	
≥**60-year group**			0.314 (0.102; 0.113 to 0.514)		.002^b^		N/A
	Knowledge	0.010 (0.151; −0.286 to 0.307)		.95		.99	
	Needs	0.386 (0.213; −0.031 to 0.803)		.07		.35	
	Cost	0.286 (0.214; −0.134 to 0.705)		.18		.91	
	Expectation	0.219 (0.160; −0.095 to 0.533)		.17		.86	
	Information	0.563 (0.130; 0.308 to 0.819)		<.001^c^		<.001^c^	

^a^N/A: not applicable.

^b^*P*<.05.

^c^*P*<.001.

**Table 3:** Relationship between gender and perception scores regarding smartphone-based hearing aid apps. The reference group was male respondents.

	Coefficient (SE; 95% CI)	*P* value	Adjusted *P* value
Knowledge	−0.053 (0.102; −0.253 to 0.147)	.61	.99
Needs	0.160 (0.170; −0.173 to 0.494)	.35	.99
Cost	0.129 (0.137; −0.140 to 0.397)	.35	.99
Expectation	−0.189 (0.120; −0.424 to 0.046)	.12	.58
Information	−0.027 (0.103; −0.229 to 0.175)	.79	.99
Total	−0.031 (0.079; −0.186 to 0.124)	.70	N/A^a^

^a^N/A: not applicable.

#### Hearing Loss and Tinnitus

We next evaluated whether subjective hearing loss or tinnitus influenced the perception of SHAA. There were significant correlations between hearing loss and the total perception score (*P*=.001). The presence of hearing loss was strongly associated with the needs category (*P*<.001), but there were no significant associations with the other categories. The degree of hearing loss indicated by the VAS score was closely related to the total scores (*P*=.001) and needs (*P*<.001; Table 4).

Although the presence of tinnitus did not show a significant association with total scores, it was positively correlated with the needs category (*P*=.003). The VAS score for tinnitus did have significant associations with SHAA perception (Table 5).

**Table 4:** Relationship between subjective hearing loss and perception scores regarding smartphone-based hearing aid apps.

Response			Coefficient (SE; 95% CI)		*P* value		Adjusted *P* value
**Subjective hearing loss (yes or no)**			0.049 (0.014; 0.021 to 0.076)		.001^a^		N/A^b^
	Knowledge	0.021 (0.023; −0.024 to 0.066)		.36		.99	
	Needs	0.266 (0.030; 0.208 to 0.324)		<.001^c^		<.001^c^	
	Cost	0.030 (0.027; −0.024 to 0.083)		.28		.99	
	Expectation	0.024 (0.021; −0.018 to 0.066)		.26		.99	
	Information	0.017 (0.015; −0.012 to 0.046)		.24		.99	
**Visual analogue scale score of hearing loss (if hearing loss present)**			0.079 (0.024; 0.031 to 0.126)		.001^a^		N/A
	Knowledge	0.045 (0.037; −0.028 to 0.117)		.23		.99	
	Needs	0.304 (0.066; 0.175 to 0.434)		<.001^c^		<.001^c^	
	Cost	0.024 (0.040; −0.054 to 0.102)		.55		.99	
	Expectation	0.059 (0.034: −0.008 to 0.127)		.08		.42	
	Information	0.057 (0.026; 0.006 to 0.107)		.03^d^		.14	

^a^*P*<.01.

^b^N/A: not applicable.

^c^*P*<.001.

^d^*P*<.05.

**Table 5:** Relationship between subjective tinnitus and perception scores regarding smartphone-based hearing aid apps.

Response			Coefficient (SE; 95% CI)		*P* value		Adjusted *P* value
**Subjective tinnitus (yes or no)**			0.003 (0.012; −0.022 to 0.027)		.82		N/A^a^
	Knowledge	0.049 (0.020; 0.011 to 0.088)		.01^b^		.06	
	Needs	−0.094 (0.027; −0.147 to −0.041)		.001^c^		.003^b^	
	Cost	−0.001 (0.024; −0.047 to 0.046)		.98		.99	
	Expectation	0.022 (0.020; −0.018 to 0.061)		.28		.99	
	Information	−0.007 (0.014; −0.034 to 0.020)		.60		.99	
**Visual analogue scale score of tinnitus (if hearing loss present)**			0.021 (0.022; −0.022 to 0.064)		.34		N/A
	Knowledge	0.068 (0.031; 0.007 to 0.128)		.03^b^		.14	
	Needs	−0.089 (0.056; −0.200 to 0.021)		.11		.57	
	Cost	0.049 (0.044; −0.037 to 0.135)		.26		.99	
	Expectation	0.052 (0.035; −0.018 to 0.121)		.15		.72	
	Information	−0.003 (0.026; −0.054 to 0.049)		.92		.99	

^a^N/A: not applicable.

^b^*P*<.05.

^c^*P*<.01.

#### Expected Price of SHAAs

**Figure 2:**
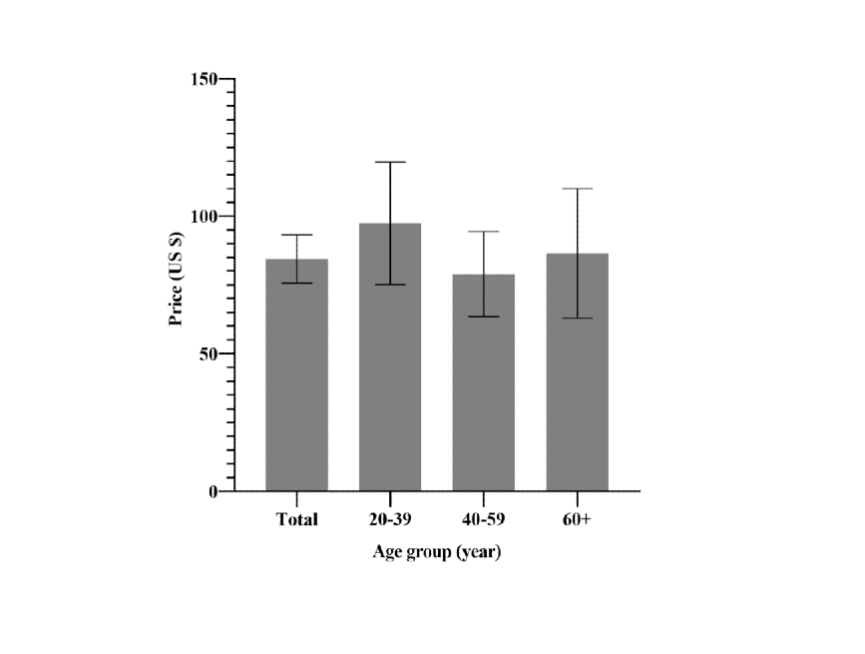
Average expected cost for smartphone-based hearing aid apps according to age group. Error bars indicate 95% CIs.

The average expected cost for an annual subscription to an SHAA was US $84.43 (95% CI 75.66-93.21). Analyzed by age group, the average expected prices were US $97.37 (95% CI 75.10-119.54), US $78.98 (95% CI 63.46-94.41), and US $86.47 (95% CI 62.91-109.93) in the 20-39–year group, the 40-59–year group, and in the ≥60-year group, respectively (Figure 2). The expected cost was significantly correlated with economic status (*P*=.02), whereas it was not significantly associated with other categories (Table 6).

The experts’ average expected cost for an annual subscription to a premium version app was US $32.48 (95% CI 17.81-47.24), and 33% (14/42) of respondents answered that the app should be available at no cost. As for an entry-version app, the expected cost for an annual subscription was US $9.69 (95% CI 2.68-16.70) on average, and 71% (30/42) of respondents expected this app to be provided free of charge.

The average cost for an annual subscription expected by potential users was markedly higher than that expected by hearing specialists (based on the premium version; *P*<.001). A total of 45% (19/42) of respondents among the potential users were not willing to pay for the app, which was much lower than the percentage of the hearing specialist group who thought the SHAA should be provided for free (*P*<.001).

**Table 6:** Factors affecting the expected price of smartphone-based hearing aid apps.

Variable			Coefficient (SE; 95% CI)		*P* value
**Age (years)**					
	40-59	−1.991 (1.348; −4.633 to 0.651)		.14	
	≥60	−1.180 (1.509; −4.137 to 1.778)		.43	
Sex (female)			0.971 (0.983; −0.955 to 2.898)		.32
**Education level**					
	High school graduate	−1.156 (1.875; −4.831 to 2.518)		.54	
	University graduate	0.122 (1.997; −3.793 to 4.037)		.95	
Economic status			−1.474 (0.638; −2.723 to −0.224)		.02^a^
Hearing loss visual analogue scale score			0.261 (0.209; −0.148 to 0.671)		.21
Tinnitus visual analogue scale score			−0.207 (0.179; −0.559 to 0.145)		.25

^a^*P*<.05.

## Discussion

### Principal Findings

This cross-sectional study recruited 261 participants, consisting of 219 outpatients and 42 hearing specialists from multiple locations in South Korea, to avoid regional bias. In addition, as non–smartphone users are not potential candidates for SHAA use, this study targeted people who own and use smartphones. Overall, only a limited number of participants had heard about SHAAs, and only 4% (9/219) of respondents were aware of the differences between SHAAs and conventional HAs. In addition, only 0.9% (2/219) of the respondents had experience using an SHAA. These results indicated an extremely low level of perception regarding SHAAs. However, it is noteworthy that 26% (57/219) of the respondents stated their intention to consider using SHAAs after they obtained information about SHAAs during the survey. This suggests that increased awareness of SHAAs may lead to their use by more individuals with hearing loss.

As the perception of SHAAs was more meaningful to people with hearing problems than to the general population, the survey was conducted for ear, nose, and throat outpatients and resulted in a relatively high proportion of participants with hearing loss or tinnitus. There was also a strong association between the perception of SHAAs and the age and degree of hearing loss. The amount of information increased with age, whereas gender showed no relationship with the information. Although the degree of hearing loss influenced the purchase of an SHAA, the level of tinnitus was not related to the perception of SHAAs. These findings suggest that the demand for hearing rehabilitation devices increases with age and the development of hearing loss, indicating the necessity of providing further relevant information to elderly individuals with hearing impairment.

The expected cost was associated with the economic status. The prices that respondents were willing to pay for SHAA were relatively high in the 20-39–year group and the >60-year group. This is perhaps because the younger generation group would like to improve their own or their parents’ hearing capacity, and the older adult group faces more inconvenience from hearing loss. In addition, price was regarded as one of the most crucial factors determining whether or not to purchase an SHAA. Respondents anticipated more advanced features with an increase in price. The average expected cost for an annual subscription to an SHAA was higher than that expected by the hearing specialists. We assumed that those with hearing impairment were willing to pay a higher price than expected by specialists because of the effect of hearing loss on their quality of life. Furthermore, the expected cost was higher than the actual price of Petralex (once-off annual cost: US $59.99 for iPhone operating system), one of the most expensive SHAAs on the market [20,29]. It is notable that only 5.9% (13/219) of respondents expected the app to be free. This suggests that potential users are willing to pay a certain amount for an SHAA with the expectation of efficacy. Nevertheless, the expected cost is substantially lower than that of commercially available HAs or PSAPs. The cost of HA fitting for a single device was US $2336 in the United States [30,31]. Moreover, OTC HAs range in price from approximately US $600 to US $1000 [32], and lower-priced PSAPs range from US $250 to US $350 [33]. The life expectancy of HAs or PSAPs is approximately 5 years. A 5-year subscription to an SHAA would be approximately US $430, which is much lower than the price of HAs and similar to that of premium PSAPs. Thus, SHAAs are likely to compete with PSAPs for market share in the future.

Smartphone-based mobile health is widely used for diagnostics and therapy [34] and also supports hearing rehabilitation. Paglialonga et al [25] investigated 200 hearing health care apps available on the market. Among these apps, the largest proportion (28%) comprised sound enhancement apps [25].

SHAAs have several advantages. First, SHAAs range in price from free to US $70, and are therefore cheaper than conventional HAs overall. SHAAs are therefore likely to substitute for traditional HAs [9]. Second, patients with hearing loss can receive a call and perform HA fitting directly with their smartphones [35]. Third, because of the convenience offered by smartphones in our daily lives, SHAAs may allow patients with hearing loss to feel free from the stigma of using HAs [22]. Finally, the advantages mentioned enable SHAAs to act as *gateway products* to more sophisticated devices, such as conventional HAs [36].

South Korea’s gross domestic product per capita is US $32,310, ranking South Korea 28th across the globe [37]. In particular, South Korea has one of the highest smartphone penetration rates, with the smallest gap among all ages (percentage of adults who own a smartphone in South Korea in 2018: 18-34–year group, 99% and >50-year group, 91%) [38]. Given that smartphone use is skyrocketing worldwide, awareness of SHAAs can increase global accessibility to HA interventions.

However, it should be noted that the effectiveness of SHAAs has not been fully proven. Amlani et al [23] recommended that SHAAs be used only as a temporary means of assistance by patients using HA. Medwetsky et al [39] reported that SHAAs improved listening performance, but test participants had only mild to moderate high frequency hearing loss. As the effectiveness of SHAAs in patients with moderate to severe hearing loss is yet to be determined, it is essential to carry out a series of well-designed studies to determine the efficacy of SHAAs in hearing rehabilitation.

### Comparison With Previous Work

Previous studies have demonstrated that SHAAs can improve hearing performance in patients with and without hearing loss [20,40]. Most previous studies compared auditory performance with conventional HAs in patients with hearing loss. They evaluated the self-reported benefits and satisfaction in a small case series in a single center [20,21]. Performance and satisfaction show wide variations according to app, operation system, and type of headphones [18]. However, previous studies did not comprehensively evaluate the awareness and associated factors of SHAAs in a cross-sectional multicenter survey. Most of the participants in our study were non-HA users. As less than half of them had subjective hearing loss, we think they could be potential candidates for SHAAs. In addition, this study showed the expectation and expected cost of HAs in smartphone users and hearing professionals. Our data showed that the price of SHAAs is underestimated and suggested an expected cost, which is useful information for mobile app users and developers.

### Strengths and Limitations

One strength of this study is that it is the first study to measure the perception of SHAAs. These findings are expected to pave the way for more surveys regarding awareness of other hearing rehabilitation devices. In addition, because of the multicenter nature of this study, our findings are generalizable to a broad population. According to a survey conducted by the government, the urban population of South Korea is approximately 90% of the total population, which is similar to the population distribution in our results (189/219, 86.3%) [41].

One limitation of this study is that because no standardized questionnaires are available to evaluate perceptions of hearing rehabilitation devices, we modified the questionnaire of a preceding study that investigated awareness about HAs [28]. As this questionnaire was not originally designed or validated to measure perceptions of SHAAs, our findings should be interpreted with caution. A well-validated survey on the perception of hearing assistant devices such as SHAAs should be developed in the future.

In addition, the participants in this study were younger than those of known typical HA seekers [10]. In addition, we did not investigate the experience of HAs because of the small number of HA users in this study. HA users may not actively seek alternative devices, and these HA users could have altered the results of the survey.

Furthermore, our findings do not provide insight into the efficacy of SHAAs in remediating hearing loss; our focus was primarily on the perception of SHAAs. Thus, clinical validation of the effectiveness of the SHAA is required [21].

### Conclusions

SHAAs are an alternative hearing rehabilitation option for smartphone users with hearing loss who have no access to appropriate hearing rehabilitation devices because of their high costs. However, the perception of SHAAs was very low. Age and degree of hearing loss were correlated with perception scores. Potential users estimated the cost of an SHAA as approximately US $86 for a 1-year subscription. Those with hearing loss and requiring hearing rehabilitation were willing to pay a higher price than what the hearing specialists expected the price to be. In addition, a higher economic status was associated with an increased willingness to pay higher prices. Considering that a large portion of respondents showed interest in SHAA after obtaining information from the survey, enhancement of perception of SHAAs is likely crucial to expand their market base.

## Appendix

Multimedia Appendix 1 Survey on the perception of hearing aid apps.

Multimedia Appendix 2 Hearing specialist’s opinion for hearing aid apps.

## Abbreviations

HA: hearing aid
OTC: over-the-counter
PSAP: personal sound amplification product
SHAA: smartphone-based hearing aid app
VAS: visual analogue scale
