# Supplementary material for: Knowledge and Expectations of Hearing Aid Apps Among Smartphone Users and Hearing Professionals: Cross-sectional Survey
Source: JMIR Mhealth Uhealth. 2022 Jan 7;10(1):e27809. doi: 10.2196/27809 (PMC8783272; doi:10.2196/27809)
Supplement: Multimedia Appendix 1 [file mhealth_v10i1e27809_app1.pdf]

Multimedia Appendix 1

Hearing Aid Applications (Hearing Aid Apps): Awareness Survey

- -

A hearing aid application (hearing aid app) in this questionnaire is not an application provided by a hearing aid manufacturer that connects to a hearing aid and increase convenience of use, but rather an application that helps the user hear sound better.

- -

Date: MM/DD/YYYY

※ Please indicate ◯ or ∨ in the following questions.

1. Date of birth; month and year only.
   (MM/YYYY)

2. What is your gender?
   ① Male ② Female

3. Where do you currently live? Please indicate city, county, or district level. (Example: Seocho-gu, Seoul)
   ( )

4. Are you living with your children?
   ① Yes ② No

5. Please indicate your education level.
   ① Junior high school graduate or less ② High school graduate ③ College graduate or higher

6. What would your rank your economic status as?
   ① High ② High-Middle ③ Middle ④ Middle-Low ⑤ Low

7. Please indicate your current job. If you are currently unemployed, please indicate your past job.
   (1) Executive (2) Experts/Professionals (3) Administrative
   (4) Customer Service Representative (5) Salesperson
   (6) Agricultural, Forestry, Fishery-related (7) Technician and Related Functional
   (8) Machinist or Assembly-related (9) Simple Labor (10) Soldier
   (11) Student (12) Housewife (13) Unemployed (14) Other: ( )

8. Have you ever been diagnosed with any of the following conditions?

(1) Diabetes: Yes / No

(2) Hypertension: Yes / No

(3) Other: (   )

9. Do you think you have a problem with hearing?

① Yes   ② No

10. (If you think you have a problem with hearing) Please mark it how serious the problem is.

|—|—|—|—|—|—|—|—|—|—|

0 1 2 3 4 5 6 7 8 9 10

(No problem at all)   (Serious problem)

11. Do you have tinnitus often?

① Yes   ② No

12. (If you have tinnitus) Please mark it how severe it is.

|—|—|—|—|—|—|—|—|—|—|

0 1 2 3 4 5 6 7 8 9 10

(No tinnitus)   (Very severe tinnitus)

13. Have you ever considered or been recommended to use assistive devices for hearing, such as hearing aids or hearing aid applications (hereinafter referred to as hearing aid apps)?

① Yes   ② No

14. Do you know what the difference is between hearing aids and hearing aid apps?

① Yes   ② No

15. Have you ever used hearing aid apps?

① Yes   ② No

If yes, how long have you used them for: (   )

Are you currently using them?   (   )

16. Does anyone in your family or an acquaintance use hearing aid apps?

① Yes   ② No   ③ I don't know

17. Have you ever heard an explanation or seen an advertisement for hearing aid apps?

① Yes ② No

18. If you were offered a hearing aid application, would you be willing to use it?

① Yes ② No ③ I don’t know

19. In order to solve the discomfort caused by hearing loss, how much are you willing to pay for hearing aid apps? Please indicate what you would be willing to pay for use of this app for a period of 1 year.

|———|———|———|———|———|

$0 $50 $100 $150 $200 $300

You can also write a specific price. ( )

※ Your perception of “Hearing Aid Applications”

Hearing Aid Applications (Hearing Aid Apps: Awareness Survey	Strongly Agree	Slightly Agree	Agree	Slightly Disagree	Strongly Disagree
	5	4	3	2	1
Hearing aid apps are one way to make soft sound audible.	5	4	3	2	1
Hearing aid apps are different from hearing aids.	5	4	3	2	1
Hearing aid apps are a type of hearing aid.	5	4	3	2	1
Hearing aid apps are an inexpensive replacement for hearing aids.	5	4	3	2	1
I have considered using hearing aids (hearing aids, sound amplifier, or hearing aid apps) because sounds are soft.	5	4	3	2	1
I have considered using hearing aids (hearing aids, sound amplifier, or hearing aid apps) because I don't understand words well even if I can hear sound.	5	4	3	2	1
Cost is the most important factor to consider when deciding whether to use a hearing aid.	5	4	3	2	1
The more expensive the hearing aid, the better it allows sound to be heard.	5	4	3	2	1
Sound might be loud if using a hearing aid app.	5	4	3	2	1
Unwanted sounds might be amplified when using a hearing aid app.	5	4	3	2	1
I have normal hearing when using a hearing aid application.	5	4	3	2	1
I can understand words when using a hearing aid application.	5	4	3	2	1
The more consistent the use of hearing aid applications, the better the outcome.	5	4	3	2	1
Hearing aid applications should be as high-powered as possible.	5	4	3	2	1
Hearing aid applications should be used when sounds are barely heard.	5	4	3	2	1
The sooner I use a hearing aid application, the better the sound gets.	5	4	3	2	1
Hearing loss might be worsened by using a hearing aid application.	5	4	3	2	1
When using a hearing aid application, it is important to use it on both ears.	5	4	3	2	1

Cronbach's Alpha

Category	Cronbach's alpha	95% Confidence interval		Cronbach's alpha	95% Confidence interval	
		Left limit	Right limit		Left limit	Right limit
Knowledge (1-4)	**0.63**	0.55	0.69	**0.53**	0.43	0.61
				**0.68**	0.61	0.74
				**0.53**	0.44	0.62
				**0.46**	0.36	0.56
Needs (5-6)	**0.90**	0.88	0.92	**0.84**	0.81	0.87
				**0.79**	0.75	0.83
Cost (7-8)	**0.59**	0.51	0.67	**0.47**	0.37	0.57
				**0.38**	0.26	0.49
Expectation (9-12)	**0.86**	0.83	0.88	**0.80**	0.76	0.84
				**0.84**	0.81	0.87
				**0.83**	0.80	0.86
				**0.80**	0.76	0.84
Information (13-18)	**0.73**	0.68	0.78	**0.68**	0.62	0.74
				**0.67**	0.60	0.73
				**0.73**	0.67	0.78
				**0.66**	0.60	0.72
				**0.72**	0.67	0.77
				**0.70**	0.64	0.75
