# Supplementary material for: Knowledge and Expectations of Hearing Aid Apps Among Smartphone Users and Hearing Professionals: Cross-sectional Survey
Source: JMIR Mhealth Uhealth. 2022 Jan 7;10(1):e27809. doi: 10.2196/27809 (PMC8783272; doi:10.2196/27809)
Supplement: Multimedia Appendix 2 [file mhealth_v10i1e27809_app2.pdf]

# Multimedia Appendix 2

## Survey: Hearing Specialist's Opinion for Hearing Aid Applications

Date: MM/DD/YYYY

※ Please indicate ◯ or V in the following questions.

1. What are you currently working for?

① University hospital

② Private clinic

③ University (Audiology, Medical engineering, etc)

④ Company

⑤ Others

2. How long have you worked in the field of audiology or otology since graduating from college?

: (            ) years

3. What is your final degree?

① Bachelor's

② Master's

③ Doctor's

4. What are the main considerations for recommending 'Hearing Aid Application'

① Price

② Basic performance (number of channel, output, gain, frequency range, etc)

③ Additional functions (noise reduction, wireless connection, bilateral communication, etc)

④ Manufacturer

⑤ Year of release

⑥ Others: (                                        )
